# Lyme Disease in Hispanics, United States, 2000–2013

**DOI:** 10.3201/eid2203.151273

**Published:** 2016-03

**Authors:** Christina A. Nelson, J. Andrew Starr, Kiersten J. Kugeler, Paul S. Mead

**Affiliations:** Centers for Disease Control and Prevention, Fort Collins, Colorado, USA (C.A. Nelson, K.J. Kugeler, P.S. Mead);; University of Virginia School of Medicine, Charlottesville, Virginia, USA (J.A. Starr)

**Keywords:** Lyme disease, Borrelia burgdorferi, surveillance, minority health, Hispanic, United States, ticks, vector-borne infections

## Abstract

Hispanics comprise a growing portion of the US population and might have distinct risk factors for tickborne diseases. During 2000–2013, a total of 5,473 Lyme disease cases were reported among Hispanics through national surveillance. Hispanics were more likely than non-Hispanics to have signs of disseminated infection and onset during fall months.

Lyme disease (LD) is caused by the spirochete *Borrelia burgdorferi*, transmitted to humans through the bite of infected *Ixodes scapularis* and *I. pacificus* ticks. Early localized infection typically manifests as erythema migrans with concomitant fever and malaise; disseminated infection can lead to facial palsy, carditis, arthritis, or neuropathy (*1*).

Outdoor workers in LD-endemic areas have increased odds of occupational exposure to ticks and a rate of LD seropositivity substantially higher than that of the general population (*2*,*3*). In the United States, Hispanics comprise 43.6% of grounds maintenance workers and 43.4% of workers in the farming, fishing, and forestry industries, potentially placing this population at greater risk for LD from occupational exposures (*4*).

An estimated 9 million Hispanics live in the 13 states with the highest reported incidence of LD, all of which are located in the Northeast, upper Midwest, and mid-Atlantic regions (*5*,*6*). Little is known, however, about the epidemiology of LD in the rapidly growing and diverse US Hispanic population. Improved understanding of LD in Hispanics could aid prevention efforts by public health practitioners and diagnosis by clinicians. The objective of this study was to describe the epidemiology of LD in the US Hispanic population and identify differences between Hispanics and non-Hispanics with LD by using national surveillance data.

## The Study

LD is a nationally notifiable condition, and cases are reported by state and local health departments to the Centers for Disease Control and Prevention (CDC) through the National Notifiable Diseases Surveillance System in accordance with previously established protocols (*7*). LD cases reported during 2000–2007 were confirmed cases only. In 2008, a revised case definition was implemented that altered the laboratory criteria and distinguished between confirmed and probable cases; cases reported during 2008–2013 included both categories (*8*).

We used 2010 US Census population data to calculate incidence rates (*5*,*9*). Weighting was applied to state- and county-specific numbers of cases to account for variations in completeness of ethnicity data. Descriptive statistics and comparisons were calculated by using SAS version 9.3 (SAS Institute, Cary, NC, USA). We compared median age of Hispanics and non-Hispanics with LD using the Kolmogorov-Smirnov 2-sample test. Risk ratios (RRs) were used to compare categorical data.

CDC human subjects review of the protocol determined it was not research involving human subjects. Thus, Institutional Review Board approval was not required.

During 2000–2013, a total of 374,338 LD cases were reported to CDC, of which 148,444 (39.7%) reports contained information about ethnicity and were included in this analysis. Among these, 5,473 (3.7%) persons self-identified as being of Hispanic ethnicity. Most (54.8%) Hispanics with LD were male; median age was 32 years (interquartile range 15–46 years).

Annual incidence of reported LD among Hispanics was 0.8 cases/100,000 persons, compared with 4.0/100,000 among non-Hispanics. During 2000–2001, Hispanics comprised 2.8% of all persons with LD, whereas during 2009–2013, Hispanics comprised 3.7%–4.9% of persons with reported LD. In comparison, the proportion of Hispanics in the US population increased slightly during this period, from 13% in 2000 to 16% in 2010 (*5*).

Although a bimodal age distribution was evident among both Hispanics and non-Hispanics with LD, the peak in children was less pronounced among Hispanics (Figure). Highest incidence among Hispanic children was in boys 10–14 years of age, whereas among non-Hispanic children, incidence was highest in boys 5–9 years of age. In adults, highest incidence among both Hispanics and non-Hispanics was in men 65–74 years of age.

**Figure:**
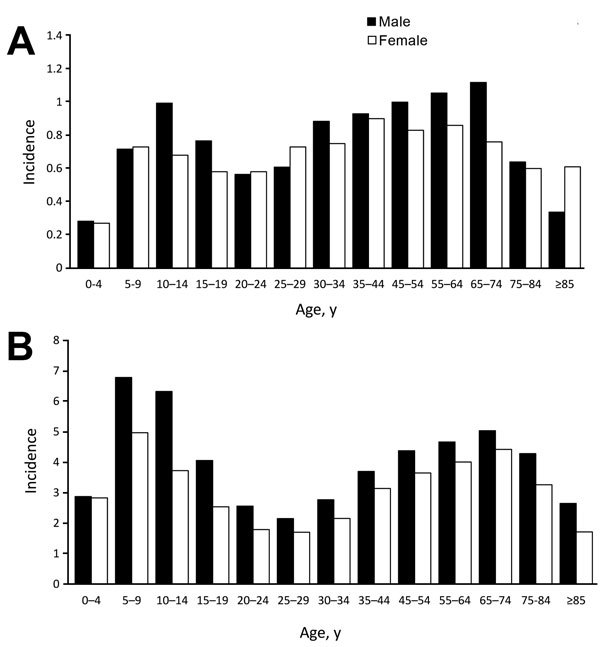
Age- and sex-specific incidence of Lyme disease among Hispanics (A) and non-Hispanics (B), United States, 2000–2013. For persons >35 years, age categories are collapsed into 10-year intervals. Incidence is cases per 100,000 persons.

Hispanics were significantly less likely than non-Hispanics to have disease onset during the summer months (RR 0.85, 95% CI 0.83–0.88) and more likely to have disease onset during the fall months (RR 1.15, 95% CI 1.07–1.24) (Table 1). Although erythema migrans was the most commonly reported clinical feature for both groups, it was less commonly reported among Hispanics than non-Hispanics (RR 0.83, 95% CI 0.80–0.86). Manifestations of disseminated disease, such as arthritis and facial palsy, were more commonly reported among Hispanics than non-Hispanics (Table 1).

**Table 1:** Demographic and clinical characteristics of Hispanics and non-Hispanics with LD, United States, 2000–2013*

Characteristic	Hispanic, n = 5,473	Non-Hispanic, n = 142,971	RR (95% CI)	p value
Male sex†	2,982 (54.8)	78,417 (55.0)	1.00 (0.97–1.02)	
Median age, y (IQR)	32 (15–46)	42 (16–58)		**0.0001‡**
Disease onset				
Total with known date of disease onset	3,826 (69.9)	116,600 (82.6)	–	
Summer months, Jun-Aug	2,170 (56.7)	77,548 (66.5)	**0.85 (0.83–0.88)**	
Fall months, Sep–Nov	637 (16.7)	16,821 (14.4)	**1.15 (1.07–1.24)**	
Clinical features				
Total with information on clinical features	2,696 (49.3)	90,180 (63.1)	–	
Erythema migrans	1,605 (59.5)	64,660 (71.7)	**0.83 (0.80–0.86)**	
Arthritis	854 (31.7)	25,647 (28.4)	**1.11 (1.05–1.18)**	
Facial palsy	391 (14.5)	7,529 (8.4)	**1.74 (1.58–1.91)**	
Atrioventricular block	36 (1.3)	952 (1.1)	1.26 (0.91–1.76)	
Meningitis	36 (1.3)	1,026 (1.1)	1.17 (0.84–1.63)	
Residence in high-incidence state§	4,937 (90.2)	130,305 (91.1)	**0.90 (0.82–0.98)**	

*Values are no. (%) unless otherwise indicated. Statistically significant differences between the comparison groups are in bold. IQR, interquartile range; LD, Lyme disease; RR, risk ratio.  †Percentage of persons with LD for whom sex is known (n = 5,442 Hispanics, n = 142,625 non-Hispanics). ‡The substantial difference in median age between the US Hispanic population (27 y) and the US non-Hispanic population (42 y) most likely accounts for the difference seen here. §Defined as 1 of the 13 highest-incidence states that accounted for 95% of all reported confirmed cases of LD in 2010: Connecticut, Delaware, Maine, Maryland, Massachusetts, Minnesota, New Hampshire, New Jersey, New York, Pennsylvania, Vermont, Virginia, and Wisconsin.

As expected, >90% of LD cases overall were reported from high-incidence states, although Hispanics with LD were slightly less likely to report residence in a high-incidence state (RR 0.90, 95% CI 0.82–0.98). All of the statistical associations were similar when analysis was restricted to confirmed cases only, with the exception of residence in a high-incidence state, which became nonsignificant (RR 0.99, 95% CI 0.89–1.10).

After weighting, nearly half of all estimated cases of LD among Hispanics were from New York or New Jersey (Table 2). Among counties with at least 75 estimated LD cases among Hispanics during the study period, highest incidence among Hispanics occurred in Columbia County, New York (170.4 cases/100,000 persons); Sussex County, New Jersey (111.4/100,000); and Hunterdon County, New Jersey (106.3/100,000).

**Table 2:** Locations with the highest number of estimated cases and incidence of LD among Hispanics, United States, 2000–2013*

Location	No. reported cases among Hispanics	% Total reported cases with ethnicity information	Estimated total no. cases†	% Total estimated no. Hispanics with LD	No. annual estimated cases/100,000 Hispanics	Counties with highest estimated incidence among Hispanics‡
All states	5,473	39.7	13,786	100	0.8	–
New York	1,825	52.8	3,456	25.1	3.6	Columbia (170.4), Putnam (61.3), Dutchess (47.4)
New Jersey	474	14.2	3,331	24.2	7.6	Sussex (111.4), Hunterdon (106.3), Warren (41.3)
Connecticut	986	50.6	1,950	14.1	14.5	Windham (45.6), New London (30.8), Fairfield (11.9)
Massachusetts	491	36.0	1,364	9.9	7.8	Plymouth (17.3), Norfolk (13.1), Middlesex (8.5)
Pennsylvania	356	28.8	1,238	9.0	6.1	Bucks (18.3), Northampton (16.3), Chester (14.3)
Maryland	253	35.7	708	5.1	5.4	Howard (16.0), Baltimore (14.1), Anne Arundel (8.8)

*LD, Lyme disease. †After correcting for missing ethnicity data. Calculated as follows: (no. reported cases)/x = (% with ethnicity information)/100, where x is the weighted number of cases. ‡Incidence calculated as number of annual estimated cases in county/100,000 Hispanic residents in county. Only counties with a substantial number of cases were included in this comparison. Seventy-five weighted cases was chosen as the cutoff based on distribution.

## Conclusions

Overall, the epidemiology of LD among Hispanics was similar to that among non-Hispanics: bimodal age distribution, slight predilection in males, and clustering in states to which LD is highly endemic were apparent (*10*). However, we identified several important differences. Most notably, Hispanics with LD were significantly more likely than non-Hispanics with LD to have signs of disseminated infection and symptom onset during fall months.

Although the overall incidence of LD in Hispanics was lower than that in non-Hispanics, additional research is needed to determine the reasons underlying these differences and the extent of any LD underdiagnosis in the Hispanic population. Inadequate healthcare access, language barriers, and lack of LD awareness could cause both underdiagnosis and delays in diagnosis in the Hispanic population. During 2009–2013, a total of 41.5% of Hispanics lacked health insurance, compared with 15.1% of non-Hispanic whites; 15.5% of Hispanics described delay in or nonreceipt of medical care because of cost (*11*). Furthermore, whether the predilection toward symptom onset in the fall months for Hispanics results from delays in medical care or other factors, such as seasonal outdoor labor patterns, is unclear. Lastly, because a larger proportion of Hispanics than the overall US population live in urban areas (*12*), the risk for LD might be differentially diluted in Hispanics.

Our findings were subject to several limitations. First, we had to exclude more than half of reported LD cases because of missing ethnicity data. Although we have no reason to believe that case reports with missing ethnicity data differed otherwise from those included in this study, we cannot exclude this possibility. Ethnicity reporting is also subject to error. Finally, surveillance data are limited by underreporting and reporting bias, which might differ by state and between Hispanic and non-Hispanic populations.

Reaching at-risk populations with culturally and linguistically appropriate prevention education is essential. Although some educational materials about prevention of tickborne diseases have been translated to Spanish (*13*,*14*), additional translations and modifications to address cultural differences would be helpful. Furthermore, targeted educational campaigns could enhance use of these materials and improve the reach, retention, and overall impact of prevention education.

We identified specific risk groups and patterns of LD within the US Hispanic population. Direct and more in-depth assessments regarding prevention practices, knowledge, and LD epidemiology on local and national scales will further the understanding of LD risk in this population and guide future targeted prevention and education efforts.
